# Integration of a menstrual health intervention in a community-based sexual and reproductive health service for young people in Zimbabwe: a qualitative acceptability study

**DOI:** 10.1186/s12913-022-07818-5

**Published:** 2022-03-30

**Authors:** Mandikudza Tembo, Jenny Renju, Helen A. Weiss, Ethel Dauya, Nancy Gweshe, Precious Ndlovu, Portia Nzombe, Chido Dziva Chikwari, Constancia Vimbayi Mavodza, Constance R. S. Mackworth-Young, Rashida A. Ferrand, Suzanna C. Francis

**Affiliations:** 1 MRC International Statistics & Epidemiology Group, London School of Hygiene and Tropical Medicine, London, UK; 2 Biomedical Research and Training Institute, Harare, Zimbabwe; 3 Clinical Research Department, London School of Hygiene and Tropical Medicine, London, UK; 4 Department of Public Health, Environments and Society, Faculty of Public Health and Policy, London School of Hygiene and Tropical Medicine, London, UK; 5 Department of Global Health and Development, Faculty of Public Health and Policy, London School of Hygiene and Tropical Medicine, London, UK

**Keywords:** Menstrual health, Acceptability, Community-based interventions, Youth, Service delivery, Service integration

## Abstract

**Background:**

Despite being fundamental to the health and well-being of women, menstrual health is often overlooked as a health priority and access to menstrual health education, products, and support is limited. Consequently, many young women are unprepared for menarche and face challenges in accessing menstrual health products and support and in managing menstruation in a healthy and dignified way. In this paper, we examine the acceptability of a comprehensive menstrual health and hygiene (MHH) intervention integrated within a community-based sexual and reproductive health (SRH) service for young people aged 16–24 years in Zimbabwe called CHIEDZA.

**Methods:**

We conducted focus group discussions, that included participatory drawings, with CHIEDZA healthcare service providers (*N *= 3) and with young women who had attended CHIEDZA (*N* = 6) between June to August 2020. Translated transcripts were read for familiarisation and thematic analysis was used to explore acceptability. We applied Sekhon’s thematic framework of acceptability that looks at seven key constructs (affective attitudes, burden, ethicality, intervention coherence, opportunity costs, perceived effectiveness, and self-efficacy). Data from FGDs and meeting minutes taken during the study time period were used to triangulate a comprehensive understanding of MHH intervention acceptability.

**Results:**

The MHH intervention was acceptable to participants as it addressed the severe prevailing lack of access to menstrual health education, products, and support in the communities, and facilitated access to other SRH services on site. In addition to the constructs defined by Sekhon’s thematic framework, acceptability was also informed by external contextual factors such as sociocultural norms and the economic environment. Providers highlighted the increased burden in their workload due to demand for MHH products, and how sociocultural beliefs around insertable menstrual products compromising virginity can negatively affect acceptability among young people and community members.

**Conclusions:**

MHH interventions are acceptable to young women in community-based settings in Zimbabwe as there is great unmet need for comprehensive MHH support. The integration of MHH in SRH services can serve as a facilitator to female engagement with SRH services. However, it is important to note that contextual external factors can affect the implementation and acceptability of integrated SRH and MHH services within communities.

**Trial registration:**

Registry: Clinicaltrials.gov, Registration Number: NCT03719521, Registration Date: October 25, 2018.

**Supplementary Information:**

The online version contains supplementary material available at 10.1186/s12913-022-07818-5.

## Background

Menstrual health and hygiene (MHH) is integral to women’s reproductive health and overall well-being and encompasses access to knowledge, materials, and facilities to manage menstruation with privacy and dignity. MHH also involves the broader psychological, environmental, and socio-political factors that inform how menstruation is managed [1, 2]. Globally, many women face challenges in managing their menstruation as MHH-related issues continue to be shrouded in secrecy and taboo and overlooked as a health priority [3]. As a result, many girls and women, particularly those in low- to middle-income countries (LMICs), lack access to MHH knowledge, products, and support, and experience anxiety, shame, and stigma as they approach menarche and throughout their reproductive years [4–7]. In LMICs such as Zimbabwe, many girls and young women are forced to either use inadequate alternatives such as tissue paper or old cloth to manage their menstruation and/or to miss school or work entirely during this time [8–10]. It is therefore critical that MHH is prioritised and addressed as a core component of women’s lives to empower women and to achieve global gender equality in accordance with the 2030 Agenda of Sustainable Development Goals [2, 11].

There is a growing body of literature looking at MHH interventions. However, programs, research, and policy continue to address MHH as a stand-alone issue and most interventions have focused on school-going girls [9, 12, 13]. A systematic review of MHH interventions in LMICs reports that sustainable and effective MHH interventions need to be comprehensive, contextually specific, and designed to address long-standing myths and misconceptions about menstruation and menstrual product use [14]. Another study adds peer support, health provider training, and education reinforcement over time as major drivers for menstrual product acceptance and MHH intervention success [15]. While there is an understanding of what works for MHH interventions and product uptake amongst girls in school, there is limited literature looking at the menstrual experiences of out-of-school young women and/or how MHH interventions inform their experiences of menstruation [9, 10, 14].

More recently, there has been a growing consensus that MHH and sexual and reproductive health (SRH) are intrinsically linked [13]. MHH is an important aspect of puberty and an access point for essential SRH information, services, and support structures that facilitate body autonomy from a young age, address reproductive health needs such as contraception and/or disease treatment, and improve women’s health outcomes over time [13, 16]. There is an opportunity to harness the intersections between MHH and SRH by using an integrated approach but there are limited data on the implementation or acceptability of interventions or services that integrate MHH and SRH [13, 16].

The aim of this study was to investigate the acceptability of a comprehensive MHH intervention integrated within a community-based SRH service in Zimbabwe.

## Methods

### Study design setting and participants

The MHH intervention is embedded within the ongoing CHIEDZA trial in Zimbabwe (clinical trials.gov: NCT03719521). CHIEDZA is a two-arm, cluster randomized community-based trial investigating the impact of the provision of HIV services integrated with a comprehensive package of SRH services (including the MHH intervention, condoms, STI management, and contraception counselling and products) for young women and men aged 16–24 years on population-level HIV virological outcomes [17]. The two-year trial is being conducted in 24 clusters (geographically demarcated areas that include a community centre and a primary health care clinic) in three provinces across Zimbabwe (Harare, Bulawayo, and Mashonaland East). In each province, eight clusters were randomised 1:1 to either receive existing routine health services (control arm) or to receive a comprehensive package of integrated HIV (including HIV testing and linkage to care and anti-retroviral therapy initiation and retention in care) and SRH services in a youth-friendly environment that included indoor and outdoor entertainment and friendly and non-judgemental delivery staff (intervention arm). All residents aged 16–24 years in the intervention clusters are eligible to access CHIEDZA services which are provided free-of-charge and available once a week, every week for the duration of the trial. The CHIEDZA services are delivered by three teams of trained CHIEDZA healthcare service providers (one team per province), each comprising two youth workers, two nurses, four community health workers (CHWs), and one counsellor. Prior to implementation, all CHIEDZA healthcare service provider teams went through a two-week training that included MHH training addressing 1) the taboo, myths, and stigma around menstruation; 2) how to use, wash, and dry the menstrual cup and how to address the issue of menstrual cup use and “virginity”; and 3) how to use, wash, dry, and store reusable pads. Teams were also provided with MHH education pamphlets (Additional Files 1 and 2) and reusable pads and menstrual cups for their own use. All training materials and the structured Manual of Operations informing the MHH intervention and CHIEDZA service delivery can be found on the CHIEDZA website [17].

In this paper, we describe a qualitative study that included members of the CHIEDZA healthcare service teams and female clients accessing CHIEDZA services across the 12 intervention clusters, conducted one year (midway) into implementation.

### The menstrual health and hygiene intervention

Formative work that included stakeholder engagement, participatory workshops, focus group discussions (FGDs), and in-depth interviews (IDIs) with CHWs, young women (aged 16–24 years old), and other key stakeholders such as the Ministry of Health and Child Care guided the development and design of the MHH intervention. Within CHIEDZA, the MHH intervention was designed to address access to pain medication, access to MHH education, support, and products, and to facilitate the de-stigmatization and taboo around menstruation. The details of the formative work will be published elsewhere.

**Fig. 1:**
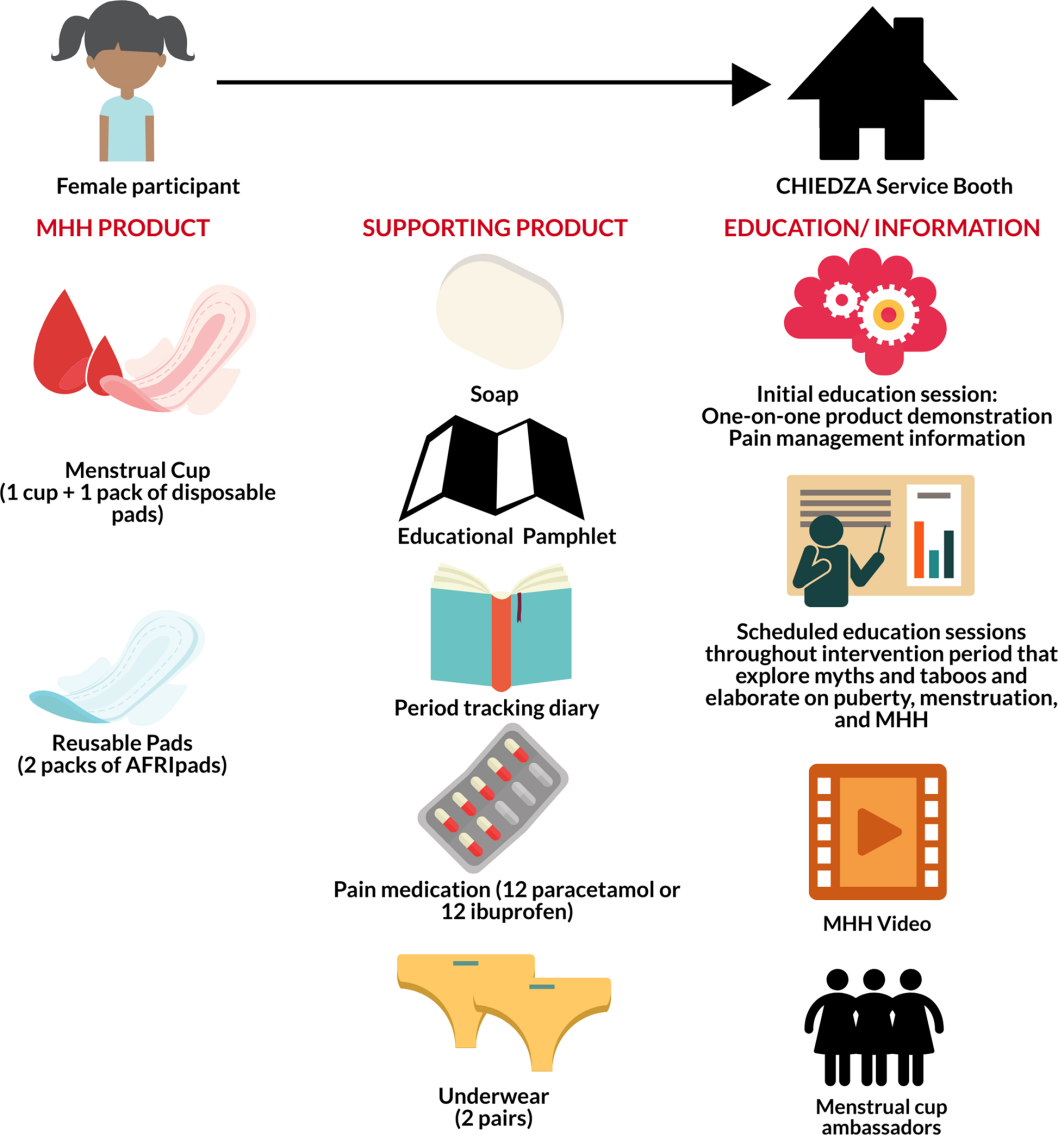
The MHH intervention in CHIEDZA

The MHH intervention was piloted from April-July 2019 in the four intervention clusters in Harare as CHIEDZA had a phased roll-out plan for intervention clusters that started with Harare province [18]. Key results from the pilot study highlighted that 1) sociocultural factors were a barrier to menstrual cup uptake; 2) environmental factors were a barrier to reusable pads uptake; 3) education for community members including caregivers and partners is key to intervention acceptability; and 4) there was a great need for MHH products and education in the community [18]. These results were used to refine and scale-up the MHH intervention across the 12 CHIEDZA intervention clusters. The MHH intervention was offered to all female CHIEDZA clients and included comprehensive MHH education and support, a simple period tracking diary, pain medication (a choice between paracetamol or ibuprofen), two pairs of underwear, soap, and a choice between reusable pads (AFRIpads that can be used for up to two years) and the menstrual cup (the Butterfly Cup that can used for up to ten years). CHIEDZA healthcare service provider teams were also joined by trained menstrual cup ambassadors to facilitate menstrual cup sensitization and promotion onsite and to provide ongoing support for new menstrual cup users (Fig. 1).

### Impact of COVID-19 on the CHIEDZA trial

By February 2020, COVID-19 was officially considered a global pandemic [19]. In response, the Zimbabwean government announced a national lockdown that commenced on March 30^th^, 2020 [20]. All CHIEDZA services were stopped at this time and recommenced on May 18^th^, 2020 in an adapted form in compliance with national COVID-19 restriction guidelines. Adaptions included: 1) mask-wearing by all providers and participants; 2) social-distancing at the CHIEDZA site; 3) removal of all social activities to discourage social gathering on site; 4) limitations on the number of participants screened and registered at any one time; and 5) limited service hours to allow for CHIEDZA intervention team members and participants to get home before the nationally stipulated curfew. These changes in the implementation of CHIEDZA across the three provinces are important to note as they removed key aspects of the youth-friendly intervention, including social spaces and activities such as pool, music, darts, and outdoor sports, that made CHIEDZA different from the standard health services and attractive to young people, especially young men in the communities.

### Study procedures

**Fig. 2:**
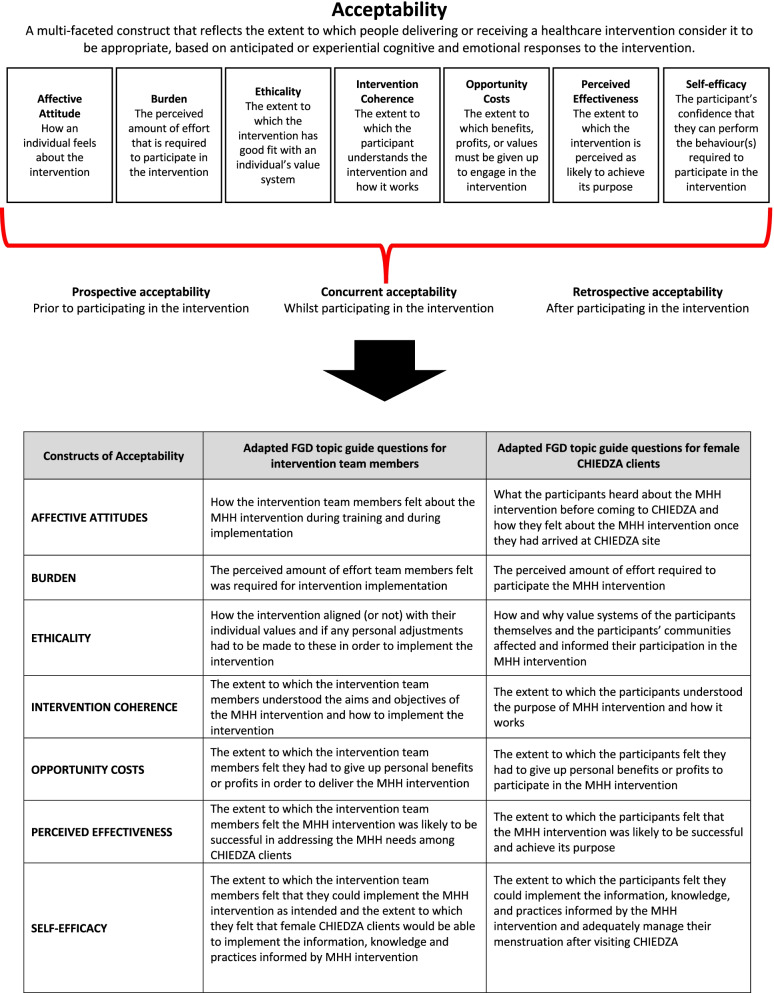
Representation of how Sekhon’s theoretical framework of acceptability (TFA) informed acceptability FGD topic guides

This qualitative study was conducted from June to August 2020 using FGDs and participatory drawings that explored how the participants viewed or perceived CHIEDZA and the MHH intervention within it. FGDs were carried out with both the CHIEDZA providers and the female participants. Semi-structured topic guides were informed by findings from the MHH intervention pilot [18] and Sekhon’s Theoretical Framework of Acceptability (TFA) that looks at seven key constructs of acceptability (affective attitude, burden, ethicality, intervention coherence, opportunity costs, perceived effectiveness, and self-efficacy) (Fig. 2) [21]. All FGDs were conducted face-to-face by NG, PNd, PNz, and three experienced female research assistants (RAs) independent from the implementation team, in either Shona, Ndebele, or English (as preferred by the participants) and took 60–75 min. Written informed consent was obtained before the FGDs were initiated and pseudonyms were used throughout to facilitate confidentiality and maintain anonymity.

### FGDs with the CHIEDZA healthcare service provider teams

Approximately one year into the two-year CHIEDZA study, one FGD was conducted with each of the three provincial CHIEDZA healthcare service provider teams. All CHIEDZA healthcare providers were asked to participate in the FGDs and were interviewed in their respective teams as this how they operated and implemented the MHH intervention in the field. One FGD had seven participants with two CHWs missing and the other two FGDs had eight participants with one nurse missing in each FGD. FGDs took place off-site, outside of CHIEDZA operating hours, and in a quiet private area to ensure confidentiality. The topic guide for the intervention team FGD explored how they perceived and experienced the MHH intervention before implementation (during training for CHIEDZA) and during implementation (Fig. 2).

### FGDs and participatory drawings with the participants

Approximately one year into the CHIEDZA study, participants were approached over a two-week period by female RAs, informed about the MHH intervention study, and asked if they were willing to be contacted via telephone to participate in the FGDs. Six FGDs were carried out with female CHIEDZA clients. Participants were purposively sampled to include 12–15 young women for each of the two FGDs per province. In each province, one FGD was among 16–19 year olds and the other one was among 20–24 year olds. Participants were purposively selected to reflect the MHH intervention uptake and MHH product choice distribution observed amongst study participants and included a range of those who did, and did not, take up the MHH intervention, and those who chose the menstrual cup or reusable pads respectively. Only the RAs were privy to participant product choice and FGD topic guide questions were kept general to prevent stigma and bias during the discussions.

The topic guide explored what participants had heard about and how they perceived the MHH intervention before coming to CHIEDZA, while at CHIEDZA, and after leaving CHIEDZA (Fig. 2). FGDs included and started with a participatory-drawing element. Done individually, in the shared FGD space, participants were asked to draw and describe CHIEDZA to further explore and understand how participants perceived CHIEDZA and the MHH intervention within it.

### Observations and note-taking from CHIEDZA intervention team meetings

To gain a deeper understanding of operational issues and the context in which the MHH intervention was being conducted, observations of the weekly interventional team meetings attended by both healthcare service providers and research team members were carried out from March 2019 to December 2020.

### Data management and analysis

FGDs were audio recorded, transcribed verbatim and then translated into English for a hybrid approach of inductive and deductive thematic analysis [22]. Initial deductive coding was based on seven constructs of the TFA, and inductive coding was used to explore other themes that were not covered by the TFA. Transcripts were read through for familiarization and coded manually by MT [23]. A senior social scientist (JR) also coded some of the initial transcripts and compared notes with MT to ensure coding consistency, comparability, and to facilitate collaborative thematic analyses throughout [24]. Transcripts were then imported to NVivo 12 software and MT organized the data into pre-defined key themes outlined by the constructs of the TFA. Coded transcripts were then discussed with and reviewed by the senior social scientist and additional themes and sub-themes were generated [25]. Data analysis of the FGDs followed Braun and Clarke’s (2006) guide to conducting a thematic analysis [23]. Themes and sub-themes were continually reviewed and refined to capture emerging new codes. Quotes were captured to highlight thematic areas and increase our understanding of the context. Thematic and visual analyses of participatory drawings, through in-depth discussions between the RAs, MT and JR, were used to reveal the more nuanced feelings and perceptions held by participants [26]. Researchers physically laid out drawings side-by-side, grouped drawings according to shared themes, and investigated and discussed emerging themes to understand the data produced by the FGD participants.

In addition to the FGDs, MT attended, collected, and reviewed detailed meeting minutes of the weekly CHIEDZA intervention team meetings [27].

Data from the FGDs, participatory drawings, and meeting minutes were triangulated to generate a comprehensive understanding of the acceptability of CHIEDZA and the MHH intervention within it among CHIEDZA healthcare service providers and clients.

## Results

Overall, 23 CHIEDZA healthcare service providers (Table 1) and 84 female CHIEDZA clients (Table 2) took part in the FGDs and shared their views and perspectives on CHIEDZA and the MHH intervention. Our findings highlight how the seven constructs of the TFA inform acceptability from the perspectives of both the providers and female CHIEDZA clients. In addition to the constructs defined by TFA, external context, including sociocultural beliefs and the economic environment, also informed the acceptability of this intervention among interview participants. In this section we detail how various constructs informed MHH intervention acceptability among both the CHIEDZA healthcare service providers and the female participants in the FGDs.

**Table 1:** FGD Participant Demographics (CHIEDZA healthcare service providers)

	**CHIEDZA healthcare service providers** *N* = **23**	
**Age Category**		*n* (%)
	18 – 25	3 (13.0)
	25 – 50	18 (78.3)
	> 50	2 (8.7)
**Sex**	Male	9 (39.1)
	Female	14 (60.9)
**Province**	Harare	8 (34.8)
	Bulawayo	7 (30.4)
	Mashonaland East	8 (34.8)

**Table 2:** FGD Participant Demographics (female CHIEDZA clients)

	**Female CHIEDZA clients** *N* = **84**	
		*n* (%)
**Age Category**	16—19	46 (54.8)
	20—24	38 (45.2)
**MHH product choice**	Reusable Pads	60 (71.4)
	Menstrual Cup	22 (26.2)
	Did not uptake MHH product	2 (2.4)
**Province**	Harare	30 (35.7)
	Bulawayo	28 (33.3)
	Mashonaland East	26 (31.0)

### Affective attitudes

Many of the female participants were “*very happy*” about the MHH intervention and appreciated the opportunity to access free menstrual health education and products. Participant drawings also reflected CHIEDZA as a fun space, with friendly “*smiling*” staff, where young women could access menstrual pads and other health services. Some of the participants described CHIEDZA as a *“safe space”* for MHH-related discussion and the MHH intervention as a great initiative for young women as it addressed their unmet need for menstrual support:

*“When I heard about CHIEDZA, I felt happy because of the products [menstrual cups/reusable pads/analgesics] they provide for girls in my community…*” (Bulawayo, FGD, 16 – 19 years old).

Similarly, many of the CHIEDZA healthcare service providers were “*very happy*” about the MHH intervention. Most team members expressed feeling *“proud”* about being able to deliver an intervention that would improve menstrual experiences for young women in the community:

*“The MHH intervention is so appreciated in these communities, a person who previously took menstrual products will just come to say thank you. So, you see that people are very appreciative and thankful”* (Harare, FGD, CHW).

### Burden

None of the participants reported that being a part of the CHIEDZA and MHH intervention within CHIEDZA was a burden and stated:

*“CHIEDZA does not force us to come every Wednesday we actually love coming here and we are not forced at all”* (Bulawayo, FGD, 16 – 19 years old).

In contrast, some service providers reported that while the MHH intervention was liked by the clients and *“a hook”* in bringing young women to CHIEDZA, implementing the intervention required additional effort and time. According to the team members, many clients required extensive menstrual health related support over time, especially those that chose to take up the menstrual cup:

*“It’s just that we get [WhatsApp] messages and calls from clients outside CHIEDZA times when we are now at home. For example, they can ask you about cups and challenges that they are facing then you have to explain to them or ask them to come back to the site”* (Bulawayo, FGD, Nurse).

### Ethicality

Several members of the CHIEDZA service provider team felt that dissemination of the menstrual cup did not align with their individual value systems. Informed by strong sociocultural beliefs, many providers were uncomfortable “*promoting*” the menstrual cup as they feared it would affect “*young women’s virginity*” and encourage sexual promiscuity:

*“I fear it will break her virginity or arouse her sensual feelings leading to her wanting to engage in sexual activities”* (Bulawayo, FGD, CHW).

Ethicality among some FGD participants was also informed by sociocultural norms and negative feedback from community members:

“*There are some who have a problem with it, they say we are teaching their kids to insert things inside their private parts”* (Bulawayo, FGD, CHW).

*“Some were complaining about the cup as they were saying giving young people menstrual cups it means they are teaching them that virginity is not important. They were really criticizing CHIEDZA and the cup…”* (Harare, FGD, 16 – 19 years old).

Despite extensive MHH training and menstrual cup sensitization, many of the service providers continued to feel conflicted about the menstrual cup. In contrast, other members mentioned that, over time, their value system shifted to being in favour of the menstrual cup. Their understanding of virginity changed and their appreciation of how the menstrual cup works grew.

*“Maybe it’s because of my training where I feel like I understand my anatomy better, I would like to believe the cup is working well for me and it does not arouse me…”* (Bulawayo, FGD, Nurse).

Others also noted that while providing insertable menstrual products did not align with their values and beliefs around virginity, they *“put [their] personal views aside”* when working as CHIEDZA implementors:

*“…at first it was difficult to talk about the issue of the menstrual cup but now because I am using it and I have enough information, I can”* (Harare, FGD, CHW).

In addition to ethical concerns around distribution of the menstrual cup, some of the intervention members mentioned feeling that CHIEDZA did not meet the needs young men in the community and that the popularity of the free menstrual products, particularly the reusable pads and the analgesics, made CHIEDZA unfairly female-focused:

*“This MHH intervention is giving us problems because boys ask us [about] how this benefits them. This program makes young men feel their needs are not prioritized since it is a pads program… Consider the plight of boys”* (Bulawayo, FGD, CHW).

Despite these value conflicts, all the female participants and CHIEDZA healthcare service providers agreed that the MHH intervention was still an important and much needed component of CHIEDZA.

### Intervention Coherence

A majority of the participants and CHIEDZA healthcare service providers appeared to have a comprehensive understanding of the MHH intervention purpose and how it worked within CHIEDZA:

*“I think MHH has been used as a program for promoting health and hygiene… We are doing sexual and reproductive health thus it plays an important role in reproductive health*” (Bulawayo, FGD, CHW).

Additionally, participants reflected a clear understanding of the different MHH intervention components, such as MHH product use and management:

*“I was also taught how to manage and take care of the reusable pads. I was told that reusable pads need to be washed thoroughly with a lot of water and soap… I was told not to use the pads while they are still wet, pads need to be dry for me to use them”* (Harare, FGD, 20 -24 years old).

### Perceived effectiveness

Almost all the female CHIEDZA participants and CHIEDZA healthcare service providers reported that the MHH intervention had effectively improved the menstrual experiences of young women in the communities. Both groups noted that the scarcity and high prices of menstrual products and analgesics in the community meant that many young women could not afford menstrual products to manage their menstruation outside of CHIEDZA and that menstruation was a *“burden”*. Due to these external conditions, many participants gave examples of how the MHH intervention in CHIEDZA had positively impacted their menstrual management and general well-being:

*“Nowadays pads are very expensive… Some girls could have been forced to go and sleep with men so as to get money to buy pads”* (Bulawayo, FGD, 16 – 19 years old).

*“The pills help us a lot especially for those with severe period pains, after taking the medication we can attend school and the boys don’t even realise that we are on our periods as we will be acting all normal”* (Bulawayo, FGD, 20 – 24 years old).

Other participants noted how the presence of MHH intervention within CHIEDZA also facilitated exposure and access to other SRH services on site:

*“What I really like about this intervention is there is also HIV testing so that you know your status. I have realised that when you come here for pads, you can actually get an HIV test”* (Harare, FGD, 16 – 19 years old).

Most of the CHIEDZA participants and CHIEDZA healthcare service providers reported that the MHH intervention was effective at reducing stigma and taboo around periods, improving MHH knowledge, and improving menstruation management among young women in the community.

### Opportunity costs

Study participants highlighted some of the costs or negative implications that came with participating in or implementing the MHH intervention. CHIEDZA healthcare service providers noted that elements of the MHH intervention, at times, overshadowed and/or interfered with the implementation of the other SRH services. Team members said that MHH intervention implementation was overly time-consuming, especially given the limited service hours due to COVID-19 related restrictions. Others reported that the overwhelming need for analgesics and menstrual health education and products in the community often resulted in: 1) more work in screening ineligible young women coming to CHIEDZA seeking menstrual health assistance; and 2) CHIEDZA being framed as an MHH service which at times impacted on take up of other services, especially in the event of stock-outs of MHH materials:

*“This week we didn’t have pads and some [participants] come to the screening area and if they hear from other clients that pads are out of stock they will literally say ‘we will come back next time when the pads are available...’It shows that, for sure, menstrual products are a need and that’s why it [the MHH intervention] is being called a ‘mini CHIEDZA’”* (Harare, FGD, Nurse).

A few participants mentioned the time spent at the site in order to participate in the MHH intervention as a burden. Here, participants noted annoyance at intervention delivery being *“very slow”* as there was nothing else to do but wait while *“the queue was taking long to move [along].”* This burden of time spent at a CHIEDZA was exacerbated by the COVID-19 restrictions; the adaptions to CHIEDZA meant limited operating hours, slower processing of clients due to social-distancing, and a lack of youth-friendly activities to participate in while clients waited for services.

### Self-efficacy

Many participants left CHIEDZA “*feeling empowered”* and confident that they would be able to adequately manage their menstruation. However, some participants reported facing difficulties managing their menstruation at home due to environmental factors such as lack of water to wash reusable pads and sociocultural factors such as a lack of support from caregivers or parents for those that chose to take up the menstrual cup. This lack of community support and its impact on participant confidence was echoed by the CHIEDZA healthcare service providers:

*“I will liken educating clients about the cup as one preaching a sermon, and then you feel like this word is for me, but on your way home you meet a friend who then diverts you from what was preached... When a client goes home with [a cup] she will hear another set of information and will be convinced to not use the cup based on the advice at home”* (Harare, FGD, CHW).

## Discussion

Overall, the MHH intervention was acceptable among female CHIEDZA clients and among both male and female intervention service providers in all three provinces. The intervention was well-received by female clients, providing young women with access to much needed pain medication and menstrual products and support, and both young men and women with MHH education that debunked harmful myths and taboos around menstruation. CHIEDZA healthcare service providers supported the intervention as it addressed an unmet MHH product and education need in the communities and attracted several female clients to CHIEDZA. These findings support similar qualitative work assessing the effectiveness and acceptability of a comprehensive MH intervention program in Uganda [28].

For most participants, the MHH intervention acceptability was heavily informed by MHH product accessibility and acceptability. Our findings add to the limited literature looking at menstrual experiences among girls in Sub-Saharan Africa (SSA) and highlights that access to comfortable and effective menstrual products is important to young women and integral to their well-being as it prevents use of ineffective alternatives and harmful practices such as transactional sex to buy MHH products [29, 30]. In contrast to other studies that look at MHH product acceptability in school-going girls [15, 31], our study highlights findings from a community-based MHH intervention where there are challenges to changing or overcoming the sociocultural barriers around “virginity” and the use of insertable menstrual products such as the menstrual cup. Despite the MHH training for the CHIEDZA healthcare service providers and the menstrual cup sensitization and support for female CHIEDZA clients, long-standing internal value systems around the concept of “virginity” and negative feedback from female clients’ caregivers and other community members once they leave the CHIEDZA sites resulted in participants opting for reusable pads as opposed to the menstrual cup. While there is limited literature looking at product choice in a community-based setting, our results are similar to those found in a study looking at the acceptability of menstrual products among women and girls in Malawi [32]. Given these findings, it is critical that MHH interventions are context-specific and adaptable to the needs and preferences of the community. Acceptable MHH interventions should not only prioritise MHH education and product dissemination, but more so informed menstrual product choice.

The MHH intervention tended to overshadow other SRH services offered by CHIEDZA and, in some instances, caused provider fatigue due to the high unmet need, which was exacerbated by the socioeconomic consequences of COVID-19 in the country [20]. Additionally, the temporary cessation of social activities within CHIEDZA, because of the COVID-19 restrictions, seemingly led to CHIEDZA becoming synonymous with MHH in the communities. In this context, MHH became the sole “*hook*” to encourage young people to visit CHIEDZA. This subsequently negatively affected male engagement and exacerbated a perception by both potential and current male clients of CHIEDZA as a female-only service. These findings build on existing evidence highlighting poor male engagement with SRH services and the need for health systems to consider men’s perceptions of health services and how this informs health-seeking behaviours [33].

Models of integrated SRH provision are designed to improve health outcomes through increased access to quality care however, these positive outcomes can come with some unintended consequences [7, 34]. A systematic review of the effects of integrated care, highlighted that many models of integration tend to focus on enhancing access to multiple services at one point or on ensuring quality service but often fail to address staff thinking and/or innovative ways of how staff work in or deliver novel intervention models [34]. For the CHIEDZA healthcare service providers, the MHH intervention was perceived to be “*a hook*” or add-on service to attract female clients to CHIEDZA and expose them to other SRH services such as HIV testing. Given this understanding, some team members perceived MHH-related work as “*extra work*” that impeded their primary duties as SRH providers. Similar findings among health care providers in Kenya demonstrate that perceptions of burden inform provider acceptability and successful integration models must also consider and address perceptions of staff about their roles and tasks, especially in settings where healthcare is often delivered through vertical programmes [35].

Our study highlights that intervention acceptability is a multi-factored measure that goes beyond just intervention uptake. A strength of this study was the assessment of acceptability from the perspectives of both the intervention clients and providers and the consideration of how contextual factors within these intervention communities informed how the intervention was implemented, perceived, and experienced. Additionally, by applying a TFA that considers both prospective and retrospective evaluations of an intervention from those that deliver and receive the intervention, our study allowed for a robust assessment of acceptability of this MHH intervention overall [21].

Importantly, Sekhon’s TFA was based on how individual value systems informed acceptability. However, our study revealed that acceptability was also informed by external factors. For example, both participants and the CHIEDZA service providers expressed how sociocultural beliefs around virginity and the insertable menstrual cup negatively informed how the intervention was perceived and received by participants and people in the community. Similar studies in the SSA region corroborate our findings and report that menstrual cup acceptability in communities is often hindered by beliefs that cup use results in the loss of virginity due to the breaking of the hymen or encourages sexual behaviour as the insertion is incorrectly thought to cause arousal [1, 18, 36]. Thus, theoretical frameworks assessing acceptability must consider how context within intervention communities affect acceptability overall, especially in community-based settings.

To our knowledge, this is the first study to investigate MHH intervention acceptability in a community-based setting in an LMIC. Using a theoretical framework of acceptability, this study provided an in-depth understanding of how both individual, community, and contextual external factors inform intervention acceptability. Our study was also conducted in a community-based setting allowing for a much needed analysis of MHH intervention acceptability outside of a school-based setting and among non-school-going women in an LMIC [37]. Our findings evidence that, when implemented outside of a school-based setting, MHH interventions should consider how external factors inform acceptability over time. Our findings also add to the guidance gap on effective and acceptable models for integrated SRH services [7, 34].

The study faced some limitations. Firstly, the qualitative data may be subject to recall bias where respondents were asked about their pre-intervention thoughts and opinions upto 12 months later and to social-desirability bias where respondents, particularly CHIEDZA service provider team members may have felt obliged to report positively on the MHH intervention and CHIEDZA. That said, our RAs were well trained to firstly disassociate themselves from the implementation team and secondly to probe for all opinions both positive and negative. Secondly, study participants did not include male CHIEDZA clients thus we do not have full understanding of intervention acceptability from the male client perspective. Thirdly, we only conducted one FGD at one time-point for each of the CHIEDZA service provider teams. While we triangulated data from the FGDs with data from the weekly CHIEDZA meetings, the small number of FGDs may have limited the depth of our findings.

## Conclusion

Overall, the study results showed that the MHH intervention itself and its integration within an SRH community-based service were acceptable among both intervention implementors and young women in communities across Zimbabwe. In an environment where there is a global push for the integration of MHH and SRH and uptake of SRH services is especially low amongst men [33], our findings add to the increasing evidence base for integrated services and provide crucial insight into some of the contextual factors policy-makers and implementors should consider and pre-emptively prepare for when designing and implementing acceptable integrated SRH services in community-based settings. Acceptable MHH interventions need to be comprehensive and need to consider the contextual factors that inform access to and informed choice of menstrual products [18, 28, 38, 39]. The integration of MHH and SRH can lead to increased female engagement with SRH services overall, however, services should consider how to meaningfully engage males and community members to ensure acceptability and effectiveness over time.

## Supplementary Information

**Additional file 1.** MHH education pamphlet for MHH intervention within CHIEDZA (front side)

**Additional file 2.** MHH education pamphlet for MHH intervention within CHIEDZA (back side)

## Data Availability

The datasets used and/or analysed during the current study are not publicly available for ethical reasons, as they contain information that could compromise the privacy and consent of the research participants. Data may be made available from the corresponding author on reasonable request.

## Abbreviations

CHW: Community Health Worker
FGD: Focus Group Discussion
IDI: In-Depth Interview
LMIC: Low- and Middle-Income Countries
MHH: Menstrual Health and Hygiene
NGO: Non-Governmental Organisation
RA: Research assistant
SDGs: Sustainable Development Goals
SRH: Sexual and Reproductive Health
SSA: Sub-Saharan Africa
TFA: Sekhon’s Theoretic Framework of Acceptability
UNICEF: United Nations Children's Fund
WASH: Water Sanitation and Health
WHO: World Health Organization
