# Supplementary material for: Integration of a menstrual health intervention in a community-based sexual and reproductive health service for young people in Zimbabwe: a qualitative acceptability study
Source: BMC Health Serv Res. 2022 Mar 30;22:421. doi: 10.1186/s12913-022-07818-5 (PMC8966602; doi:10.1186/s12913-022-07818-5)
Supplement: Supplementary file 1 — Additional file 1. MHH education pamphlet for MHH intervention within CHIEDZA (front side) [file 12913_2022_7818_MOESM1_ESM.pdf]

UNDERSTANDING YOUR PERIOD

•What is a period?
Menstruation, or a period, is a very natural process that physically mature females go through every month. During menstruation, a female will typically bleed through her vagina for about 3 to 7 days.

•How do periods happen?
Every month, a sexually mature female's ovary releases an egg cell that travels towards the uterus through the fallopian tube. The uterus prepares for a possible pregnancy by developing a uterine lining made of tissue and blood vessels. This lining is called the endometrium.

If the egg is not fertilized and pregnancy does not occur, the uterine lining is shed and it exits the body through the vagina as a mixture of blood and tissue over a course of 3 to 7 days. This cycle is called a menstrual cycle and is generally 25 to 35 days long. The cycle starts over again with an egg that begins to mature in one of the ovaries.

PRODUCTS & SERVICES

Period Products
You can choose one of the following reusable products:

- The Menstrual Cup
- Reusable Pads
- Period Pants

Period Services

- Menstrual Health education
- Pain management education
- Pain management medication (Paracetamol and/or Ibuprofen)

PERIOD PANTS

·How to use them
You should aim to change your period pants 2-3 times a day. When you feel the need to change your period pants, remove them and rinse with cold water.

After rinsing, wash them separately or with the rest of your laundry. If you are not at home when you change your underwear, fold the used period pants and place in a waterproof bag or plastic bag and wash when you get home. When washing your pants, it is very important that you use cold water and that you do not use bleach or fabric softener.

To dry the period pants, simply hang them to dry under the sun and then store away with the rest of your underwear.
Never wear your period pants when they are not completely dry.

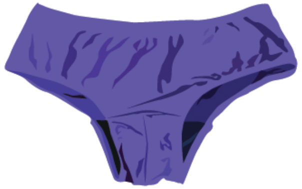

You can use your period pants for up to 2 years or until you feel they do not absorb enough anymore.

REUSABLE SANITARY PAD

·How to use one
Wash the pad before first use with cold water and soap. Dry the pads in the sun. You should aim to change your pad 2-3 times a day. Once used, fold the used pad following the 'easy fold & carry' instructions. The blood will be 'packed' inside the leakproof fabric and others will not be able to smell the pad. The plastic bag serves as extra protection.

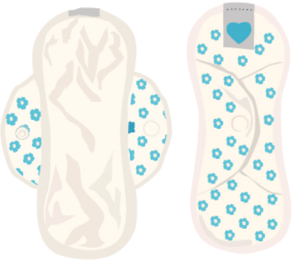

Once used, rewash and reuse. Compare it to washing knickers and wearing them again.

The project will only replace the reusable pads every 12 months, unless damaged or lost. You need to bring in the reusable pads to show that they are damaged.

MENSTRUAL CUP

·How to use one
Fold the cup as you insert it into your vagina. When removing, remember to squeeze the bottom part of the cup until you feel or hear the suction release. Then, gently rock the cup from side to side while pulling down. Make sure that you do not pull the cup out by the stem alone! It is important to relax.

When you have removed your cup, empty it into the toilet, and rinse it with water. If you do not have access to water, you can wipe it with some tissue or simply reinsert it directly after emptying it. Every month, make sure you boil your cup for 3-5min at the end of your period.

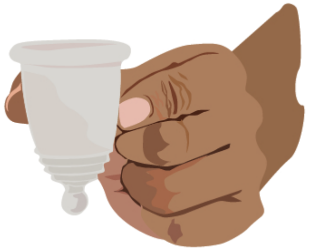

The project will only provide the cup once. It will only be replaced if it is lost or damaged.

MANAGING YOUR MENSTRUAL HEALTH

•Taking care of your body
·Tracking your period
This is an easy way to predict how long you will bleed for during your period and when you will next start your period.

This can help you prepare for your upcoming period every month and also alert you if you have missed your period (which may be a sign that you may be pregnant).
