# Supplementary material for: Integration of a menstrual health intervention in a community-based sexual and reproductive health service for young people in Zimbabwe: a qualitative acceptability study
Source: BMC Health Serv Res. 2022 Mar 30;22:421. doi: 10.1186/s12913-022-07818-5 (PMC8966602; doi:10.1186/s12913-022-07818-5)
Supplement: Supplementary file 2 — Additional file 2. MHH education pamphlet for MHH intervention within CHIEDZA (back side) [file 12913_2022_7818_MOESM2_ESM.pdf]

Here are some easy steps to follow so you can track your period using a simple calendar, diary, or piece of paper:

To measure the length of your period

Step 1: Mark down the first day that you bleed.
Step 2: Mark down the last day that you bleed.
Step 3: Repeat steps 1 and 2 for 3 months.
Step 4: Add the number of days you bleed for month 1, 2, and 3 and divide by 3 to calculate the average length of your period.

To measure your period cycle

Step 1: Write down the first day that you bleed.
Step 2: Write down the first day of your NEXT period.
Step 3: Count the number of days in-between the first days of your period for the two months.
Step 4: Estimate the first day of your next period (3rd month) by counting the same number of days between the first day of the 1st month and the first day of the 2nd month

Happy & Healthy During Your Period

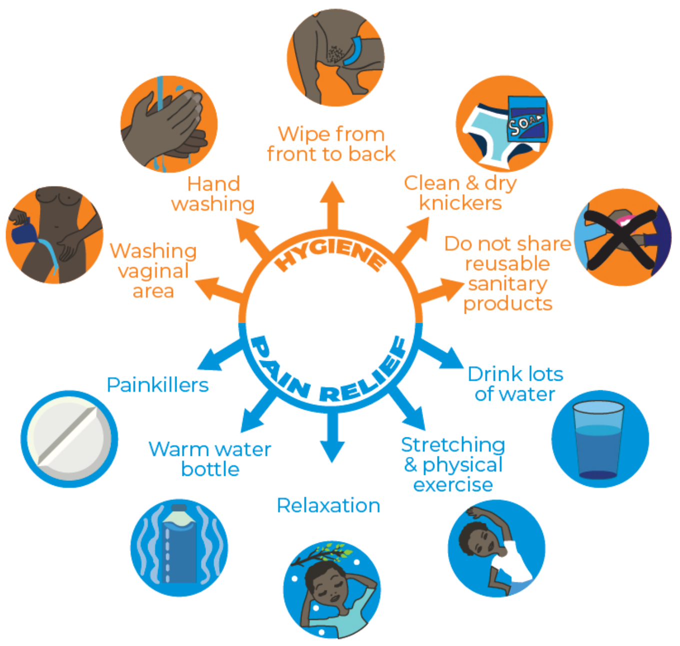

·Pain Management

If you have menstrual cramps, take some pain medication such as ibuprofen or paracetamol. For the best results, try to take these medications the day before your period is due to start or just as soon as bleeding or cramping starts.
If you do not want to or cannot use medication, heat can also help. Place a hot towel or hot water bottle on your lower back or tummy. You can also do some light stretches or other forms of exercise to relieve the pain.

Everything you need to know about...

YOUR PERIOD!

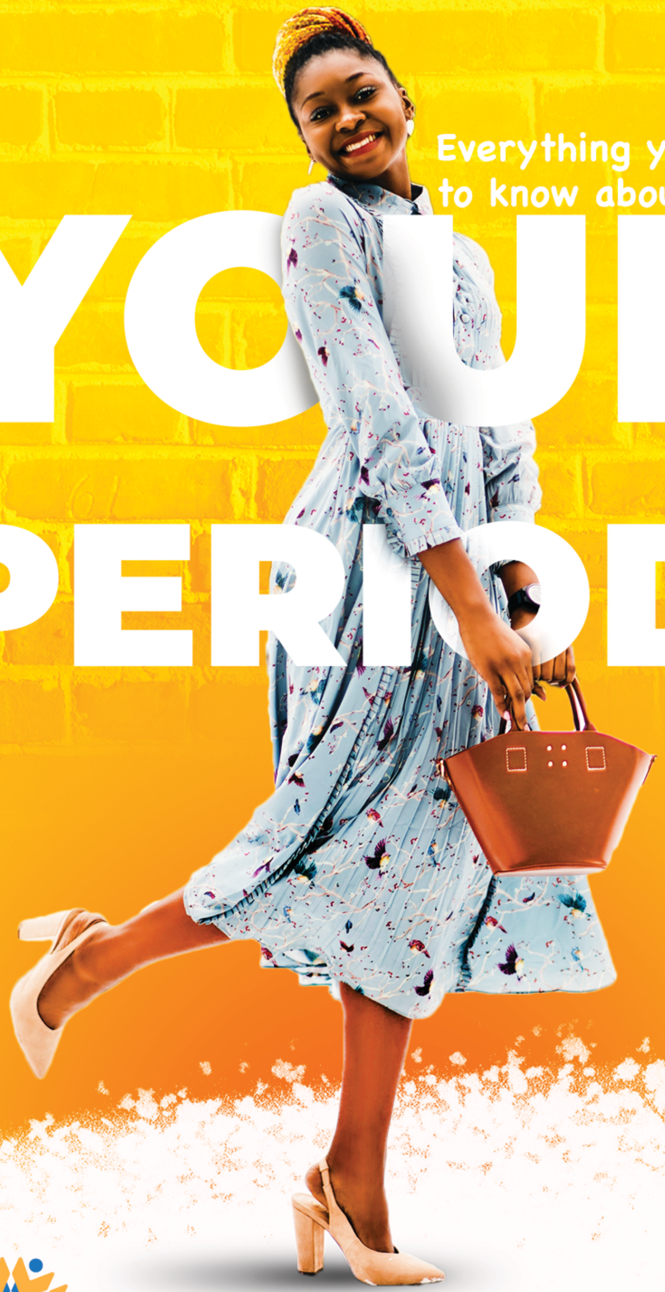

Chiedza Ukukhanya
Health For Our Future

www.chiedza.co.zw
Harare: 0779 620 908
Mashonaland East: 0716 318 730
Bulawayo: 0716 318 734
